# Risk assessment of macrovascular and microvascular events in patients with type 2 diabetes by analyzing the amplitude variation of the fourth harmonic component of radial pulse wave

**DOI:** 10.14814/phy2.14252

**Published:** 2019-10-07

**Authors:** Kuo‐Meng Liao, Chi‐Wei Chang, Sheng‐Hung Wang, Yi‐Ting Chang, Ying‐Chun Chen, Gin‐Chung Wang

**Affiliations:** ^1^ Division of Endocrinology & Metabolism of Zhongxiao Branch of Taipei City Hospital Taipei Taiwan; ^2^ MiiAnn Medical Research Center Taipei Taiwan; ^3^ Biostatistics Johns Hopkins Bloomberg School of Public Health Baltimore; ^4^ JinMu Health Technology Taipei Taiwan

**Keywords:** Harmonic analysis, macrovascular events, microvascular events, pulse wave analysis, radial pulse wave, risk factor

## Abstract

This investigation explored the hypothesis that whether the coefficient of variation of the fourth harmonic amplitude of the radial pulse wave (C4CV) predicts the risk of macrovascular and microvascular events in patients with type 2 diabetes mellitus (T2DM). Radial pulse wave and brachial blood pressure were measured at baseline in 2324 patients with T2DM and C4CV was calculated using the Fourier series method. Macrovascular and microvascular events during follow‐up were determined by medical records. We plotted the Kaplan–Meier curve and performed a Cox proportional hazard model and a log‐rank test to estimate the effectiveness of C4CV as a risk predictor. We divided patients into quartile groups based on C4CV (<4.3%, 4.3% to 6.8%, 6.8% to 11.4%, and >11.4%). Compared with patients with C4CV < 4.3%, patients with C4CV> 11.4% had a double incidence of macrovascular events (hazard ratio, 2.13; 95% CI, 1.70–2.67) and microvascular events (hazard ratio, 2.08; 95% CI, 1.67–2.58), and the incidence of cardiovascular death was three times (hazard ratio, 3.03; 95% CI, 1.10–8.83). The Cox regression analysis demonstrated that the risk of both macrovascular and microvascular outcomes increases with the increase in quartile level of C4CV value (*P* < 0.0001). These associations remained after adjustment for age, gender, smoking, systolic blood pressure, diastolic blood pressure, dyslipidemia, diabetes duration, Hba1c, and cardiovascular disease (*P* < 0.0001). C4CV is a novel independent predictor of cardiovascular mortality, macrovascular events, and microvascular events in patients with T2DM.

## Introduction

Harmonic analysis of arterial pressure pulse provides insightful hemodynamic information into the condition of the ventricular‐arterial system through the resonance effects (Wang et al. 1991; Wang and Wang 2018). Some researchers showed that changes in blood flow (Young et al. 1989; 1992; Chen et al. 1993) and function (Liao et al. 2018, 2018a,b) of an organ could be revealed by changes in arterial pulse waveforms. Therefore, harmonic characteristics derived from arterial pulses can be used to monitor the progression of cardiovascular disease. Guo et al. found that the variation coefficient (CV) of the fourth harmonic (C4) of arterial pulse waves rose sharply about half an hour before death in rats (Kuo et al. 2005). In a further clinical study, they also found that coefficient of variation of the fourth harmonic amplitude of the radial pulse (C4CV) in cancer patients was significantly higher than healthy control group, and C4CV would be further elevated before the death of the cancer patients (Kuo et al. 2004). They proposed that the C4CV is an indicator to assess the instability of the fourth harmonic component, which is one of the signs for failure of the circulatory system (Kuo et al. 2004, 2005). Thus, we tried to use this technique and assess whether larger C4CV contributes to vascular risk in patients with type 2 diabetes (T2DM). Our cohort study on T2DM manifested that larger C4CV is associated with renal dysfunction (Liao et al. 2018). Another study found that larger C4CV is associated with myocardial ischemia and a reduced left ventricular ejection fraction (<50%) (Liao et al. 2018). Recently, our preliminary study suggested that larger C4CV is independently associated with future major adverse cardiovascular events (MACE) in asymptomatic patients with T2DM (Liao et al. 2019). Therefore, we propose that C4CV may represent an integrated index of vascular structure and function in the arterial system, independent of other standard risk factors such as blood pressure, dyslipidemia, and glycated hemoglobin (Hba1c). To validate this hypothesis in this prospective cohort study, we sought to determine whether C4CV is an independent predictor of macrovascular and microvascular complications in patients with T2DM in the general clinical setting.

## Material and Methods

### Study population

Patients with T2DM and participating in the Diabetes Management Program were recruited from the Taipei City Hospital Zhongxiao Branch from 2017 to 2019. The cohort included 1370 men (59%) and 954 women (41%). The study was conducted in accordance with the Helsinki Declaration and Good Clinical Practice Guidelines. All participants signed a written informed consent form approved by the Taipei City Hospital Institutional Review Board (IRB number: ISRCTN14306167). Patients received an average of 1.8 ± 0.5 years of follow‐up. Patient demographics, baseline medical conditions, and disease prevalence are assessed by medical records and confirmed by drug use or a medical examination (Table 1). The established cardiovascular disease is defined by the history of myocardial infarction, angina pectoris, stroke, cerebral ischemia, or any revascularization procedure.

**Table 1:** Baseline clinical characteristics of patients with type 2 diabetes by C4CV quartiles

Clinical characteristics	Quartile of C4CV			
	<4.3%	4.3–6.8%	6.8–11.4%	>11.4%
*N*	581	581	581	581
Male (%)	63	61	58	54
Age, year	60 ± 12	61 ± 12	62 ± 11	65 ± 12
BMI, kg/m^2^	27 ± 4	27 ± 5	27 ± 5	27 ± 5
Waist circumference, cm	92 ± 10	93 ± 12	93 ± 12	94 ± 12
Smoke, %	15	17	15	15
SBP, mmHg	127 ± 11	127 ± 11	127 ± 11	129 ± 13
DBP, mmHg	74 ± 7	74 ± 8	75 ± 7	74 ± 9
Hba1c,%	7.0 ± 1.1	7.1 ± 1.1	7.0 ± 1.2	7.1 ± 1.1
EGFR, mL/min per 1.73 m^2^	90 ± 30	88 ± 30	87 ± 33	78 ± 35
LDL, mg/dL	86 ± 27	84 ± 26	87 ± 31	85 ± 28
HDL, mg/dL	50 ± 16	50 ± 16	51 ± ± 16	50 ± 15
TC, mg/dL	160 ± 33	158 ± 31	162 ± 36	160 ± 35
TG, mg/dL	133 ± 101	129 ± 79	133 ± 81	131 ± 92
Heart rate, beats/min	74 ± 13	72 ± 12	71 ± 13	71 ± 13
ABI	0.98 ± 0.27	0.97 ± 0.29	0.95 ± 0.30	0.95 ± 0.27
Duration of diabetes, years	10 ± 8	10 ± 7	10 ± 7	11 ± 8
Metformin,%	93	89	88	83
Beta blocker,%	14	11	14	17
Calcium blocker,%	27	32	35	40
ACE inhibitor,%	1	1	3	2
ARB,%	48	49	52	58
Statins,%	77	77	79	80
History of cardiovascular disease				
Myocardial infarction (%)	2	4	3	7
Stroke (%)	1	1	1	2
Coronary artery disease (%)	1	1	1	3

SBP, Systolic blood pressure; DBP, Diastolic blood pressure; Hba1c, Glycated hemoglobin; LDL, low‐density lipoprotein cholesterol; HDL, high‐density lipoprotein cholesterol; TC, Total Cholesterol; TG, triglycerides; EGFR, Estimated glomerular filtration rate; ACE inhibitor, angiotensin‐converting‐enzyme inhibitor; ARB, Angiotensin receptor blocker; C4CV, The coefficient of variation of the fourth harmonic amplitude of the radial pulse wave; AB, ankle–brachial index.

### Study design

The RPWT2DM collaborative project is an ongoing prospective observational study focusing on the relationship between arterial pulse waves and cardiovascular outcomes in T2DM patients. This sub‐study further focused on C4CV, a risk indicator firstly proposed by Wang et al. (Kuo et al. 2004, 2005), and its effects on macrovascular and microvascular events. All subjects underwent radial pulse wave measurements and recorded C4CV values at baseline. Radial pulse waves were noninvasively measured by medical‐grade devices that had been found to be highly reproducible (Chang and Wang 2015), and the intra‐observer and inter‐observer reliabilities met the criteria for medical interpretation (Portney and Watkins 2009; Chang et al. 2015). The standard operating procedures for environmental temperature control and radial pressure wave measurement were as described in previous studies (Chang et al. 2015, 2018). Before the measurement started, each patient was asked to rest for more than 5 min, keeping blood pressure as stable as possible. If any difference in pressure level between two consecutive foot points of pressure pulses was above the one‐fourth of pulse pressure (peak‐to‐peak pressure), the measurement is deemed as a failure of measurement. The patient will be asked to rest for 5 more minutes and then underwent radial pulse measurement again. These procedures will be repeated until the above criteria are met. With the Fourier series method (O'Rourke 1982; Katznelson 2004), the mean of fourth harmonic amplitude (C4_avg_) and the variation coefficient of four harmonic amplitude (C4CV) are defined by the following equation:

(1) $$C4_{\text{avg}}=\frac{1}{M}\sum_{m=1}^{M}C4_{m}=\frac{1}{M}\sum_{m=1}^{M}\frac{A_{4,m}}{A_{0,m}}$$

(2) $$A_{4,m}e^{-j\theta_{4,m}}=\frac{2}{L}\sum_{k=1}^{L}x_{m}\left(k\right)e^{-j2\pi \frac{k*2}{N}}$$

(3) $$\sigma_{c4}=\sqrt{\frac{\sum_{m=1}^{M}{\left(\frac{A_{4,m}}{A_{0,m}}-C4_{\text{avg}}\right)}^{2}}{M-1}}$$

(4) $$C4\text{CV}=\frac{\sigma_{c4}}{C4_{\text{avg}}}$$

where *A*_4,_ *_m_* and *θ*_4,_ *_m_* are the absolute amplitude and phase of the fourth harmonic of the *m*th radial pulse in the 12‐sec radial pulse measurement. *A*_0,_ *_m_* is the average of the *m*th radial pulse. *x_m_* (*k*) is the *k*th discrete sampled data point in the *m*th pulse signal. *L* is the total number of data points in *x_m_* (*k*), and *M* is the total number of pulses in one measurement. We divided the enrolled patients into quartile groups based on C4CV value (<4.3%, 4.3% to 6.8%, 6.8% to 11.4%, and >11.4%).

After completing the radial pulse wave measurement, blood pressure and heart rate were measured by an automatic blood pressure monitor (HBP‐9020, Omron, Japan). This blood pressure measurement is assisted by a trained operator and instructions, and there are no doctors and nurses to avoid the white coat effect.

### Outcomes

All subjects were involved in the diabetes management program and visited the study hospital every 4–6 months, conducting a clinical assessment of cardiovascular risk based on risk factors and cardiovascular events. The clinical assessment determined all the important vital status, function and activity limitation of subjects, and record all outpatient cardiovascular events. We documented all new occurrences of macrovascular and microvascular events after the baseline radial pulse wave measurement during the follow‐up period. Date of death was verified and the cause of death was confirmed by death certificates and medical record. These clinical outcomes were extracted by the IT department of the research hospital using the diabetes management project database and verified by independent monitors.

The primary interest outcomes were the composite endpoints of macrovascular events, consisting of MACE (composites of nonfatal myocardial infarction, nonfatal stroke, hospitalization for heart failure, and cardiovascular death), coronary artery disease, and severe peripheral artery disease. The endpoint of coronary artery disease was defined as a new diagnosis of single coronary artery stenosis (≥70%), multiple coronary stenoses (≥50%), and ischemic myocardium (≥10%) for further cardiac examination. Definition of nonfatal myocardial infarction, hospitalization for heart failure, and diagnosis of coronary artery stenosis was detailed in previous studies (Chang et al. 2018). The secondary interest outcomes were the composite endpoints of the microvascular events, composed of the major adverse kidney events (composite endpoints of double serum creatinine, end‐stage renal disease, kidney failure, and renal cause of death), macroalbuminuria, retinopathy, and polyneuropathy.

### Statistical analysis

Patients were classified into four groups based on the quartile of C4CV value (<4.3%, 4.3% to 6.8%, 6.8% to 11.4%, and >11.4%), with the lowest quartile (C4CV < 4.3%) serving as a reference group. Survival curves for the four C4CV groups were plotted using the Kaplan–Meier method. In Figure 2A and B, the cumulative incidence of Kaplan–Meier curves for the composite endpoints of macrovascular and microvascular was plotted, respectively, according to the quartile level of C4CV. Figure 3A–G show the cumulative incidence of Kaplan–Meier curves for each subtype of cardiovascular events separately, from the first to fourth quartile levels of C4CV. The log‐rank test was used to compare the significant difference between survival curves. We then established a Cox proportional hazard model using the survival time of the first relevant endpoint to assess the association between C4CV and the outcome of interest. The unadjusted hazard ratio (HR) and 95% confidence intervals of all outcomes, jointly and separately, in each quartile were reported (Table 2). Statistical tests of quartile linear trends were performed by Cox regression analysis. Only the first event of the relevant result was included in each Cox regression analysis. All traditional risk factors were considered in the multivariate analysis, including age, gender, smoking, systolic blood pressure, diastolic blood pressure, dyslipidemia, duration of diabetes, Hba1c, and history of cardiovascular disease. The history of cardiovascular disease was defined as a history of adverse cardiovascular events (myocardial infarction or stroke) or coronary artery disease. A value of *P* < 0.05 was considered to be statistically significant. Matlab version 9.2 for Windows (MathWorks Inc) was used for all statistical analyses.

**Figure 1:**
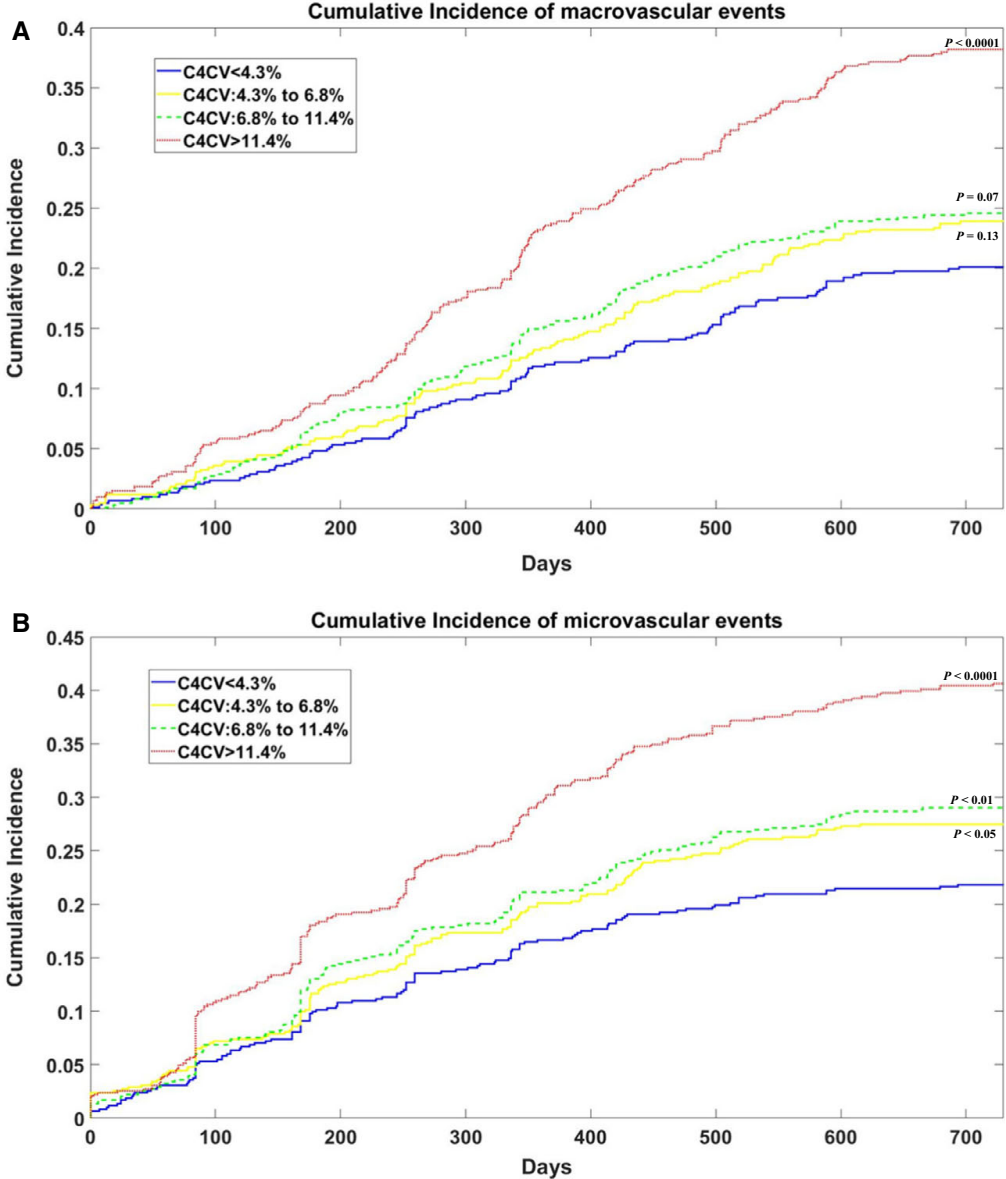
The calculation of C4CV using a typical 12‐sec radial pulse wave, noninvasively recorded by a pressure sensor at a sampling rate of 400 Hz.

**Figure 2:**
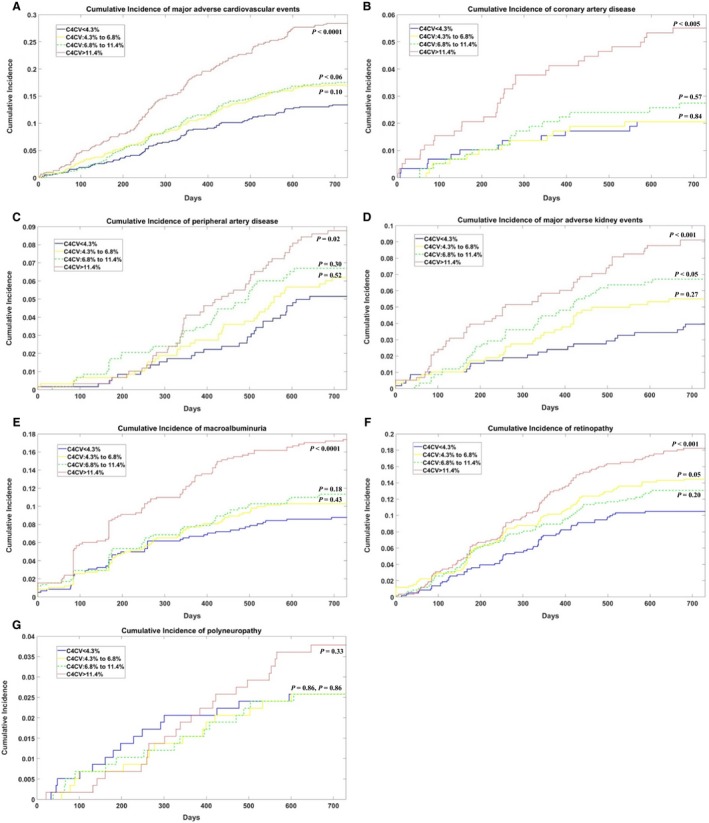
Kaplan–Meier event rates of the composite endpoints of (A) macrovascular and (B) microvascular events. Macrovascular events combined the MACE, coronary artery disease, and severe peripheral artery disease. Microvascular events combined the major adverse kidney events, macroalbuminuria, retinopathy, and polyneuropathy (*N* = 2324); *P* values were the result of the log‐rank test. The reference group for the log‐rank test is the smallest quartile of C4CV (<4.3%).

**Figure 3:**
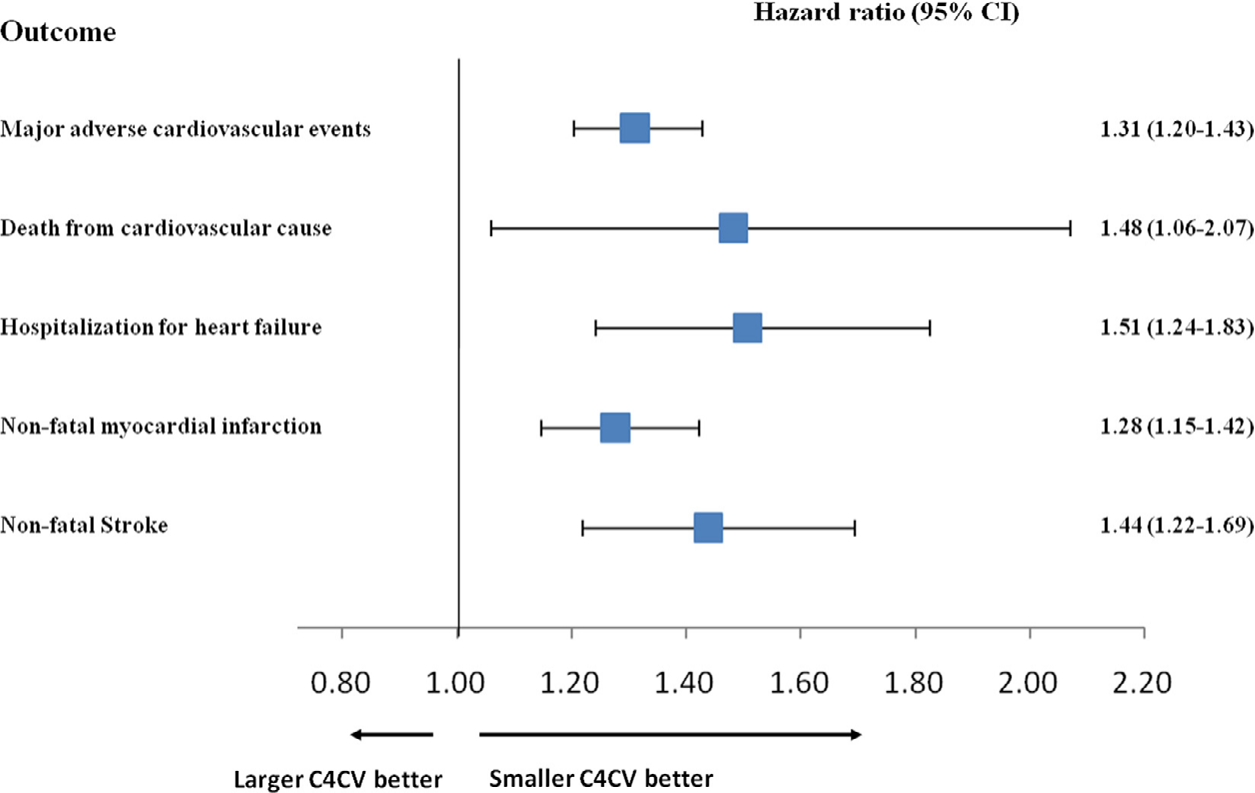
Kaplan–Meier event rates of (A) MACE, (B) coronary artery disease, (C) severe peripheral artery disease, (D) major adverse kidney events, (E) Macroalbuminuria, (F) Retinopathy, and (G) Polyneuropathy (*N* = 2324); *P* values were the result of the log‐rank test. The reference group for log‐rank test is the smallest quartile of C4CV (<4.3%).

**Table 2:** Effects of the coefficient of variation of the fourth harmonic amplitude of the radial pulse (C4CV) on all clinical outcomes in 2324 type 2 diabetic patients

	Quartile of C4CV					
	<4.3	4.3–6.8	6.8–11.4	>11.4	*P* for trend	*P* for trend*
*n*	581	581	581	581		
Macrovascular events						
No. events	117	139	143	222		
Hazard ratio (95% CI)	1.00	1.22 (0.951–1.55)	1.27 (0.991–1.62)	2.13 (1.70–2.67)	<0.0001	<0.0001
Major adverse cardiovascular events						
No. events	78	99	102	165		
Hazard ratio (95% CI)	1.00	1.3 (0.97–1.75)	1.34 (1.00–1.80)	2.31 (1.77–3.03)	<0.0001	<0.0001
Coronary artery disease						
No. events	12	12	16	32		
Hazard ratio (95% CI)	1.00	1.00 (0.45–2.22)	1.33 (0.631–2.82)	2.71 (1.4–5.26)	<0.001	<0.01
Severe peripheral artery disease						
No. events	30	36	39	51		
Hazard ratio (95% CI)	1.00	1.21 (0.74–1.96)	1.32 (0.82–2.13)	1.74 (1.11–2.72)	<0.05	<0.05
Microvascular events						
No. events	127	160	169	236		
Hazard ratio (95% CI)	1.00	1.30 (1.03–1.64)	1.38 (1.1–1.74)	2.08 (1.67–2.58)	<0.0001	<0.0001
Major adverse kidney events						
No. events	23	32	39	53		
Hazard ratio (95% CI)	1.00	1.4 (0.82–2.40)	1.72 (1.03–2.88)	2.37 (1.45–3.87)	<0.001	<0.01
Macroalbuminuria						
No. events	51	60	66	101		
Hazard ratio (95% CI)	1.00	1.18 (0.81–1.72)	1.31 (0.91–1.88)	2.07 (1.48–2.90)	<0.0001	<0.01
Retinopathy						
No. events	61	84	76	106		
Hazard ratio (95% CI)	1.00	1.41 (1.01–1.96)	1.27 (0.904–1.77)	1.81 (1.32–2.48)	<0.001	<0.05
Polyneuropathy						
No. events	15	15	15	22		
Hazard ratio (95% CI)	1.00	1.00 (0.49–2.04)	1.00 (0.49–2.04)	1.47 (0.76–2.83)	0.25	0.65

The reference group for hazard ratio is the first quartile of C4CV in the descending order (<4.3).

* *P* for trend controlling for age, sex, smoking, systolic pressure, dyslipidemia, duration of diabetes, EGFR, Hba1c, and history of cardiovascular disease.

## Results

Table 1 shows the baseline clinical characteristics of 2324 patients with T2DM, with 59% of men and 16% of smokers. The mean age and duration of diabetes were 62 ± 12 and 10 ± 8 years, respectively. Over a mean follow‐up of 1.8 years, 621 macrovascular events and 692 microvascular events occurred. Among them, 444 cases had MACE. We divided patients into quartile groups based on C4CV levels at baseline and thus the remaining analysis is focused on the C4CV quartile (<4.3%, 4.3–6.8%, 6.8–11.4%, >11.4%). The mean values of systolic and diastolic blood pressure were not significantly different across the four groups. The mean values of the ankle–brachial index, body mass index, waist circumference, smoking proportion, cholesterol level, and Hba1c level were also not significantly different across the four groups. Patients with the largest quartile of C4CV (>11.4%) had an average age increase of 4 years and average estimated glomerular filtration rate (EGFR) decreased by 13%, compared with the other three groups (Table 1) .

### C4CV and macrovascular events

Table 2 showed that the quartile C4CV level was related to the risk of the macrovascular outcome. Based on the four quartiles of C4CV, the incidence of macrovascular events increased from 20% to 24% to 25% to 38%, and its Kaplan–Meier curve is shown in Figure 2A. The log‐rank test showed a significantly higher incidence of macrovascular events in the highest quartile compared to the lowest C4CV quartile (*P* < 0.0001), and its hazard ratio (HR) is 2.13 (95% CI, 1.70–2.67). After adjustment for age, gender, smoking, systolic blood pressure, diastolic blood pressure, dyslipidemia, diabetes duration, Hba1c, and cardiovascular disease, C4CV remained significantly associated with composite endpoints of macrovascular disease (*P* < 0.0001).

We took further steps to analyze the incidence and HR of MACE, jointly and separately. Figure 3A showed the cumulative incidence of MACE by a Kaplan–Meier curve. The incidence of MACE in patients with C4CV > 11.4% was more than double that of patients with C4CV < 4.3% (log‐rank test *P* < 0.0001). Compared with the lowest C4CV quartile, the HRs of MACE were 1.30 (95% CI, 0.97–1.75), 1.34 (95% CI, 1.00–1.80), and 2.31 (95% CI, 1.77 ‐ 3.03) for the second, third, and fourth quartile of C4CV, respectively (Table 2). In a detailed evaluation of MACE, the quartile of C4CV was significantly associated with nonfatal stroke, nonfatal myocardial infarction, heart failure, and cardiovascular death (Figure 3). This association was still significant after adjusting for age, gender, smoking, systolic blood pressure, diastolic blood pressure, dyslipidemia, diabetes duration, EGFR, Hba1c, and cardiovascular disease history (*P* < 0.0001 for nonfatal stroke and myocardial infarction, *P* < 0.001 for heart failure, and *P* < 0.05 for cardiovascular death, respectively).

**Figure 4:**
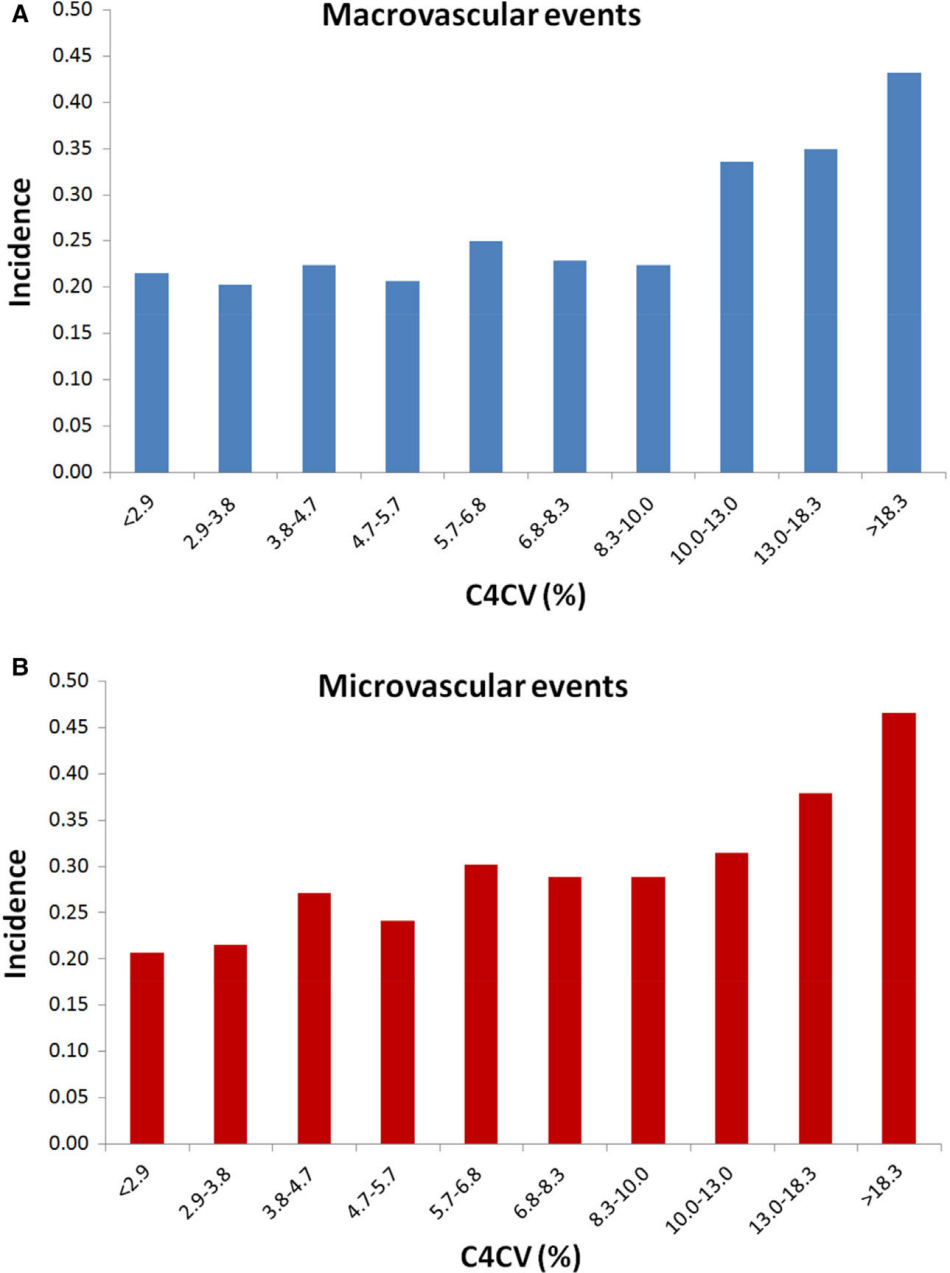
Effects of baseline C4CV quartile (<4.3%, 4.3–6.8%, 6.8–11.4%, and >11.4%) on MACE.

In evaluations of other macrovascular events, patients with the highest quartile C4CV also had the highest incidence of coronary artery disease and peripheral arterial disease (Fig. 3B and C); the log‐rank tests were *P* < 0.001 and *P* < 0.05, respectively. The Cox proportional hazard model demonstrated that the C4CV quartile was independently associated with coronary artery disease and peripheral arterial disease after adjustment for age, gender, smoking, systolic blood pressure, diastolic blood pressure, dyslipidemia, diabetes duration, EGFR, Hba1c, and cardiovascular disease history (Table 2).

### C4CV and microvascular events

The secondary survey was whether the quartile C4CV predicts a new occurrence of microvascular complications. Figure 2B showed that as the C4CV quartile increases, the cumulative incidence of composite microvascular endpoints increases from 22% to 28% to 29% to 41%. The log‐rank test showed that those incidences of microvascular events in the second, third, and fourth quartiles of C4CV were significantly higher than the lowest C4CV quartile (*P* < 0.05, *P* < 0.01, and *P* < 0.0001, respectively). The Cox proportional hazard model further demonstrated that quartile level of C4CV independently predicted (HR, 1.26; 95% CI, 1.18–1.35 per quartile increase) the composite endpoint of microvascular events after adjusting for age, gender, smoking, systolic blood pressure, diastolic blood pressure, dyslipidemia, diabetes duration, EGFR, Hba1c, and cardiovascular disease history.

When we observed each of the microvascular diseases in Figure 3D–G, the cumulative incidence of all listed events in C4CV > 11.4% was higher than in patients with C4CV < 4.3%, and HRs were listed as follows: major adverse kidney events (HR, 2.37; 95% CI 1.45–3.87), macroalbuminuria (HR, 2.07; 95% CI, 1.48–2.90), retinopathy (HR, 1.81; 95% CI, 1.32–2.48), and polyneuropathy (HR, 1.47; 95% CI, 0.76–2.83). Cox regression analysis showed that quartile C4CV was associated with a new incidence of each microvascular complication except for polyneuropathy, both before and after controlling for age, sex, smoking, systolic pressure, dyslipidemia, duration of diabetes, Hba1c, and history of cardiovascular disease (Table 2).

## Discussion

These data demonstrate that C4CV is associated with macrovascular events, microvascular events, and cardiovascular mortality in diabetic populations, independent of the traditional risk factors described above. Compared with patients with the lowest C4CV quartile, patients with the highest C4CV quartile had a two‐ to threefold increase in the risk of MACE, coronary artery disease, major adverse kidney events, macroalbuminuria, and cardiovascular death, and a more than 70% increased risk of severe peripheral arterial disease and retinopathy. These significant results manifested that the C4CV quartile provides independent predictive value for cardiovascular complications except for polyneuropathy, in patients with T2DM. The first quartile (<4.3%) and the fourth quartile (>11.4%) of C4CV were significantly different in age, gender ratio, and EGFR (*P* < 0.001 for age and EGFR using the *t* test; *P* < 0.001 for the gender ratio using the Mann–Whitney *U* test). Therefore, we build a multivariate Cox proportional hazard model to adjust those confounders (age, gender ratio, and EGFR) as well as the traditional risk factors including, smoking, systolic blood pressure, diastolic blood pressure, dyslipidemia, diabetes duration, Hba1c, and cardiovascular disease history. The results confirmed that the quartile level of C4CV is independently associated with the risk of macrovascular events (adjusted HR, 1.19; 95% CI 1.11–1.28; per quartile level increase) and microvascular events (adjusted HR, 1.17; 95% CI 1.09–1.25; per quartile level increase). The results also demonstrated that the quartile level of C4CV was independently associated with the risk of major adverse kidney events (adjusted HR, 1.22; 95% CI 1.05–1.46; per quartile level increase). This suggested that hemodynamic status has an additional impact on the risk of future adverse kidney events, independent of current renal function indicators such as EGFR.

The C4CV will be increased by about 20% in dying rat after a fatal dose of urethane injection (Kuo et al. 2005). A study by Kuo et al found that C4CV in patients who died of cancer would reach 40%, indicating the importance of its physiological significance (Kuo et al. 2004). However, the clinical inference of the mechanism of this phenomenon has not been fully examined and elaborated. This study found for the first time that an increase in C4CV, from less than 4.3% to more than 11.4%, was associated with a significant increase in the risk of macrovascular and microvascular events. In previous studies, we found that increased C4CV is an important sign of myocardial ischemia and is associated with lower ventricular ejection fraction (Liao et al. 2018). Therefore, it can be inferred that C4CV might also be associated with coronary artery disease and heart failure. The results of this study confirm this hypothesis, demonstrating that quartile level of C4CV is positively associated with coronary stenosis and myocardial perfusion defects (unadjusted HR, 1.46; 95% CI, 1.17–1.81 per C4CV quartile increase) and can predict the risk of myocardial infarction (unadjusted HR, 1.28; 95% CI, 1.15–1.42 per C4CV quartile increase) and hospitalization for heart failure (unadjusted HR, 1.51; 95% CI, 1.24–1.83 per C4CV quartile increase). In a previous survey, we also demonstrated the feasibility of quartiles C1 and C4 as potential risk marker for MACE (Chang et al. 2019, 2019) and cardiovascular cause mortality (Liao et al. 2019). A reasonable question is whether C1 and C4CV are two harmonic characteristics controlled by the same mechanism. Therefore, we further established a multivariate Cox proportional hazard model, including the quartile level of C1 and the quartile level of C4CV. The results indicate that the quartile level of C1 and the quartile level of C4CV are two independent risk markers for macrovascular and microvascular events (*P* < 0.001, respectively, for each composite endpoints). In addition, we used a logistic regression to confirm the correlation between the ankle–brachial index < 0.9 and the two harmonic risk markers (quartile C1 and C4CV). The results showed that quartile C1 was associated with ankle–brachial index < 0.9 (*P* = 0.005), whereas quartile C4CV was not associated with ankle–brachial index < 0.9 (*P* value = 0.15). Those result suggests that C1 and C4CV may reflect cardiovascular risk for different reasons and mechanisms.

We propose that increasing C4CV may be a sign that the fundamental frequency of the arterial system does not match the heart rate. Contracting the heart at the fundamental frequency of the arterial system will preserve maximum energy in the ventricle‐arterial system (Wang et al. 2004; Lin Wang et al. 2007, 2015) and achieve the highest efficiency of perfusion of the organs (Wang and Wang 2013, 2014, 2018). This way of working will reduce the burden on the heart and use the least power to maintain the blood supply to the body, which will manifest with the optimal harmonic amplitude and the smallest coefficient variations in these harmonics (Lin Wang et al. 2015; Lin Wang and Wang 2015). However, the progression of aging atherosclerosis and cardiovascular disease will alter the perfusion status and elasticity of the macrovascular bed or microvascular bed, leading to deviations in specific frequency characteristics of the organs (Jan et al., 2003; Hsu et al. 2006). These cumulative effects will reflect changes in the overall frequency characteristics of the arterial system, which increases the difficulty for the heart to achieve frequency matching and work in the most efficient way, with evidence on the increased coefficient of variation of the fourth harmonic amplitude (Table 2). In addition, coronary atherosclerosis and stenosis leading to myocardial ischemia will lead to perfusion defects, thereby weakening the function of the heart and reducing the ability of the heart to achieve heart rate adaptation. This will increase the chance and extent of frequency mismatch between the heart and arterial systems and is also reflected in the increase in C4CV. In summary, the changes in the physical properties of the entire ventricular‐arteries system with the progression of cardiovascular disease are the main reasons why C4CV can measure cardiovascular risk and predict the incidence of cardiovascular events.

Patients with T2DM have a higher prevalence of cardiovascular disease, and the risk of macrovascular and microvascular events is two to four times higher than that of healthy people (Kannel and McGee 1979; Susan van et al. 2010; Association and A. D. 10, 2019; Association and A. D. 11, 2019). The phenomena described in the previous paragraph change the physical properties of the entire ventricular‐arterial system and increase the burden on the heart, which is difficult to fully observe in traditional risk factors. Traditional risk factors such as cholesterol levels, blood glucose levels, and albumin‐creatinine ratio are biochemical indicators. On the other hand, systolic blood pressure, diastolic blood pressure, and aortic pulse wave velocity are indicators of the physical properties of the aorta. None of them directly measured the physical characteristics of the entire ventricle‐arterial system. Lin et al. used a biomechanical model to explain why harmonic analysis of radial pulse waves is one of the complete methods for assessing the physical properties of the ventricular‐arterial system, and why these harmonic indicators of arterial pulse can reflect the function of the heart and organs (Lin Wang et al. 2015; Wang and Wang 2018; Lin Wang 2019). Our previous study found that traditional cardiovascular risk factors have limitations in identifying patients with T2DM who is at high risk of heart disease, especially in those without angina symptoms or history of cardiovascular disease.(Chen et al. 2018, 2018) Scognamiglio et al. have also shown that myocardial perfusion defects and significant coronary stenosis in a great portion of asymptomatic patients with T2DM cannot be identified by traditional risk factors (Scognamiglio et al. 2006). Those who fail to be identified early in cardiovascular disease will result in a poor prognosis and may face MACE or even cardiovascular death before appropriate medical intervention. Therefore, screening the radial pulse and C4CV could improve the cardiovascular risk stratification and enhance the identification in the early stage of cardiovascular disease. This will contribute to the quality of life in patients with T2DM by early, additional cardiovascular examination and active treatment.

**Figure 5:**
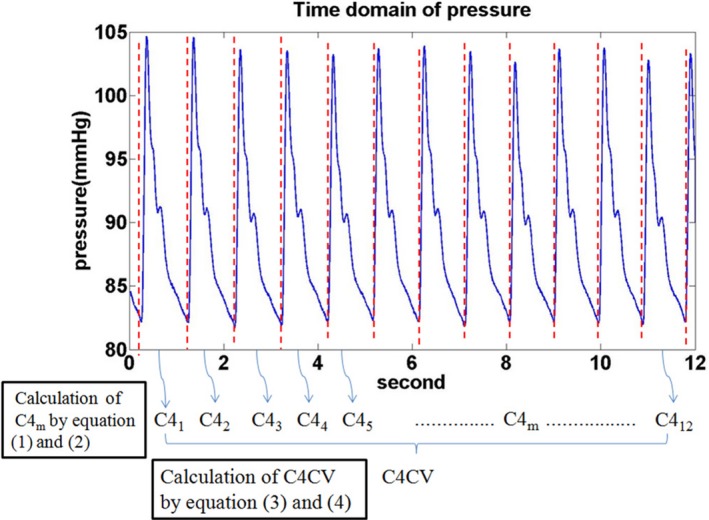
Incidence of (A) macrovascular and (B) microvascular events according to the decile level of C4CV values. Macrovascular events combined the MACE, coronary artery disease, and severe peripheral artery disease. Microvascular events combined the major adverse kidney events, macroalbuminuria, retinopathy, and polyneuropathy (*N* = 2324).

To the best of our knowledge, this study demonstrated for the first time that C4CV can independently predict the risk of macrovascular and microvascular events. Therefore, we sought to further determine a C4CV threshold that is significantly associated with future cardiovascular events. We plotted the Figure 5, which showed the incidence of macrovascular and microvascular events according to the decile level of C4CV values. As shown in the C4CV defined in the study design and as shown in Figure 5, C4CV is a value for measuring the instability of the fourth harmonic of the radial pulse wave. Cox proportional hazard model further demonstrated that the largest three decile level of C4CV had significant increasing risk for macrovascular events. Cox proportional hazard model further demonstrated that the first three largest decile levels of C4CV have a significantly increased risk of macrovascular events, comparing to the smallest decile levels of C4CV (HR, 1.69, 95% CI, 1.19–2.41 for C4CV between 10.0%‐13.0%; HR, 1.76, 95% CI, 1.24–2.50 for C4CV between 13.0% and 18.3%; HR, 2.36, 95% CI, 1.68–3.31 for C4CV > 18.3%). The Cox model also demonstrated that the first three largest decile levels of C4CV have a significantly increased risk of microvascular events, comparing to the smallest decile levels of C4CV (HR, 1.64, 95% CI, 1.14–2.35 for C4CV between 10.0%‐13.0%; HR, 2.00, 95% CI, 1.40–2.84 for C4CV between 13.0% and 18.3%; HR, 2.64, 95% CI, 1.88–3.71 for C4CV > 18.3%). Since the incidence of macrovascular and microvascular events is significantly increased when C4CV is above 10%, we believe C4CV > 10% may be a reasonable criterion for further cardiovascular examination in patients with T2DM.

Despite significant results, the study has some limitations. The primary objective of the study was to demonstrate a significant independent association between C4CV and future risk of macrovascular and microvascular events in patients with T2DM. However, as an observation cohort study, a summary of causal relationships or mechanisms between clinical variables is limited. Therefore, including noninvasive radial pulse measurements in a well‐defined cardiovascular treatment studies is a good way to discover the underlying mechanisms of C4CV changes and how they affect the risk of future cardiovascular events. It will also help us gain insight into how the arterial system works and how to extract useful information from arterial pressure waves and help us monitor the progression of cardiovascular disease.

In conclusion, the quartile level of C4CV independently predicts the risk of macrovascular and microvascular events in patients with T2DM. Patients with the highest quartile of C4CV had the highest incidence of macrovascular (38%) and microvascular diseases (41%) during an average of 1.8 years of follow‐up. The results suggest that C4CV is a powerful independent predictor of most cardiovascular complications except for polyneuropathy, in patients with T2DM, both with and without an established cardiovascular disease. Because the radial pulse wave measurement and harmonic analysis are simple, noninvasive, and relatively inexpensive, C4CV may become a first‐run check technique for assessing cardiovascular risk in routine clinical practice of T2DM.

## Conflict of Interest

The authors declare that they have no competitive interests.
