# Erratum

**DOI:** 10.14814/phy2.14311

**Published:** 2019-12-13

https://doi.org/10.14814/phy2.14252

In Chang et al. (2019), the following errors were published on page 3, 4, 5, 8, and 9

Figure Captions

1. Figure 1. The calculation of C4CV using a typical 12‐s radial pulse wave, noninvasively recorded by a pressure sensor at a sampling rate of 400 Hz.

2. Figure 2. Kaplan–Meier event rates of the composite endpoints of (a) macrovascular and (b) microvascular events. Macrovascular events combined the MACE, coronary artery disease, and severe peripheral artery disease. Microvascular events combined the major adverse kidney events, macroalbuminuria, retinopathy, and polyneuropathy (*N* = 2324); *p* values were the result of the log‐rank test. The reference group for the log‐rank test is the smallest quartile of C4CV (<4.3%).

3. Figure 3. Kaplan–Meier event rates of (a) MACE, (b) coronary artery disease, (c) severe peripheral artery disease, (d) major adverse kidney events, (e) Macroalbuminuria, (f) Retinopathy, and (g) Polyneuropathy (*N* = 2324); *p* values were the result of the log‐rank test. The reference group for log‐rank test is the smallest quartile of C4CV (<4.3%).

4. Figure 4. Effects of baseline C4CV quartile (<4.3%, 4.3–6.8%, 6.8–11.4%, and >11.4%) on MACE.

5. Figure 5. Incidence of (a) macrovascular and (b) microvascular events according to the decile level of C4CV values. Macrovascular events combined the MACE, coronary artery disease, and severe peripheral artery disease. Microvascular events combined the major adverse kidney events, macroalbuminuria, retinopathy, and polyneuropathy (*N* = 2324).

The figure captions were published in wrong order and should instead be the following:

Figure 1. Kaplan–Meier event rates of the composite endpoints of (a) macrovascular and (b) microvascular events. Macrovascular events combined the MACE, coronary artery disease, and severe peripheral artery disease. Microvascular events combined the major adverse kidney events, macroalbuminuria, retinopathy, and polyneuropathy (*N* = 2324); *p* values were the result of the log‐rank test. The reference group for the log‐rank test is the smallest quartile of C4CV (<4.3%).

Figure 2. Kaplan–Meier event rates of (a) MACE, (b) coronary artery disease, (c) severe peripheral artery disease, (d) major adverse kidney events, (e) Macroalbuminuria, (f) Retinopathy, and (g) Polyneuropathy (*N* = 2324); *p* values were the result of the log‐rank test. The reference group for log‐rank test is the smallest quartile of C4CV (<4.3%).

Figure 3. Effects of baseline C4CV quartile (<4.3%, 4.3–6.8%, 6.8–11.4%, and >11.4%) on MACE.

Figure 4. Incidence of (a) macrovascular and (b) microvascular events according to the decile level of C4CV values. Macrovascular events combined the MACE, coronary artery disease, and severe peripheral artery disease. Microvascular events combined the major adverse kidney events, macroalbuminuria, retinopathy, and polyneuropathy (*N* = 2324).

Figure 5. The calculation of C4CV using a typical 12‐s radial pulse wave, noninvasively recorded by a pressure sensor at a sampling rate of 400 Hz.

The publisher apologizes for the errors.
